# Topoisomerase 2 Is Dispensable for the Replication and Segregation of Small Yeast Artificial Chromosomes (YACs)

**DOI:** 10.1371/journal.pone.0104995

**Published:** 2014-08-12

**Authors:** Jorge Cebrián, Estefanía Monturus, María-Luisa Martínez-Robles, Pablo Hernández, Dora B. Krimer, Jorge B. Schvartzman

**Affiliations:** Department of Cell and Molecular Biology, Centro de Investigaciones Biológicas (CSIC), Madrid, Spain; University of Oklahoma, United States of America

## Abstract

DNA topoisomerases are thought to play a critical role in transcription, replication and recombination as well as in the condensation and segregation of sister duplexes during cell division. Here, we used high-resolution two-dimensional agarose gel electrophoresis to study the replication intermediates and final products of small circular and linear minichromosomes of *Saccharomyces cerevisiae* in the presence and absence of DNA topoisomerase 2. The results obtained confirmed that whereas for circular minichromosomes, catenated sister duplexes accumulated in the absence of topoisomerase 2, linear YACs were able to replicate and segregate regardless of this topoisomerase. The patterns of replication intermediates for circular and linear YACs displayed significant differences suggesting that DNA supercoiling might play a key role in the modulation of replication fork progression. Altogether, this data supports the notion that for linear chromosomes the torsional tension generated by transcription and replication dissipates freely throughout the telomeres.

## Introduction

Andrew Murray and Jack Szostak [1] constructed the first Yeast Artificial Chromosome (YAC) soonafter Szostak and Blackburn succeeded to clone yeast telomeres on linear plasmid vectors [2]. This was the beginning of a crucial series of experiments that ultimately led to a whole new field: Molecular Cytogenetics. Although it was early recognized that small YACs are less stable than natural yeast chromosomes and buildup stability as their size increases [3], they do replicate and segregate. In his Nobel Prize Lecture, Szostak describes the revealing observation that indicated he succeeded to obtain the first YAC: “*When DNA molecules are separated by gel electrophoresis, circles generate a series of bands corresponding to monomers and multimers, and relaxed and supercoiled forms, leading to a complicated pattern. Linear DNA molecules don’t have any of those alternative forms, so they migrate as a single band. The two possible results of the DNA analysis were therefore quite distinct. When I analyzed the DNA from the transformants that I had recovered, about half of them contained plasmid DNA that migrated as a single band on the gel. This was perhaps the most clear-cut experiment I have ever done. It was immediately obvious that the experiment had worked, and that the Tetrahymena ends were able to act as functional telomeres in yeast*” [4]. ¿Why does small linear YACs appear as a single band when separated by gel electrophoresis? - Probably because they do not support supercoiling. Then, the crucial question is: ¿Are small linear YACs devoid of supercoiling *in* *vivo* or only when all proteins have been removed? It could be that DNA helical tension dissipates through the chromosomal ends *in* *vivo* indicating that telomeres are topologically open [5]. Replication-induced topological stress is influenced by chromosome length [6]. Therefore, although topoisomerase activity is essential as a swivel for DNA replication and transcription of genomic DNA [7], it is possible that small YACs wouldn’t need topoisomerases to replicate and segregate. To check whether or not small linear YACs require topoisomerase 2 to replicate and segregate here we constructed small circular and linear yeast minichromosomes and used them to transform yeast strains lacking DNA topoisomerase 2 (Topo2). Cells were synchronized at the beginning of the S-phase and high-resolution two-dimensional (2D) agarose gel electrophoresis was used to examine minichromosome’s replication intermediates (RIs) and segregation products in the presence and absence of Topo 2. The results obtained indicated that Topo 2 is dispensable for the replication and segregation of small linear YACs.

## Results

### Rationale

**Figure 1:**
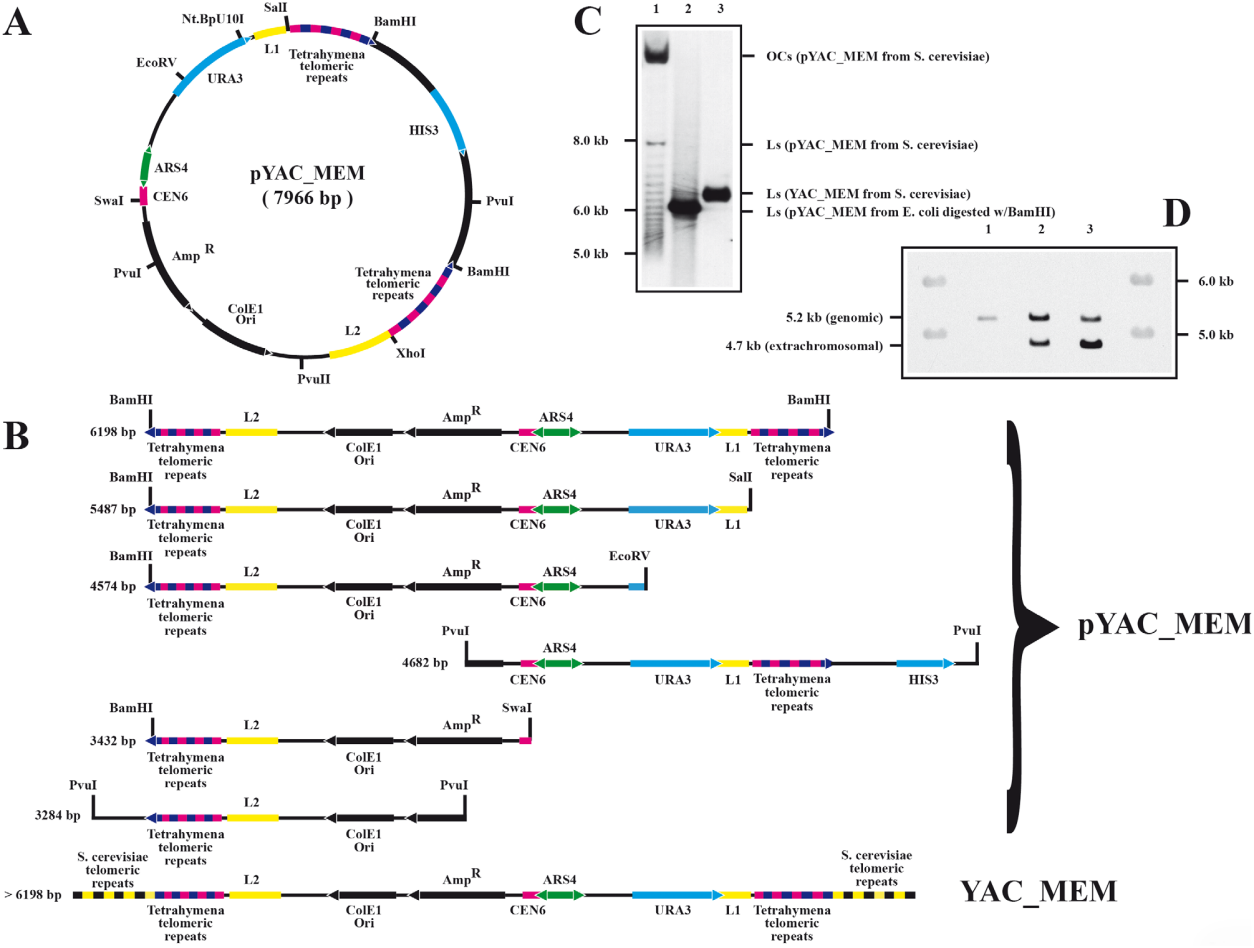
Construction of pYAC_MEM and YAC_MEM. A: Name, mass and genetic map of the circular minichromosome. The relative positions of its most relevant features are indicated inside: The centromeric sequence CEN6, the autonomous replication sequence (ARS4), URA3, the lambda DNA marker sequence (L1), *Tetrahymena* telomeric repeats, HIS3, the lambda DNA marker sequence (L2), the ColE1 unidirectional origin (ColE1 Ori) and the ampicillin-resistance gene (AmpR). Outside, the relative positions of sites recognized by specific restriction endonucleases are indicated. B: The corresponding linear maps of the circular minichromosome’s restriction fragments and their sizes are indicated. At the bottom the genetic map of YAC_MEM. C: Circular and linear DNAs analyzed in unidirectional gel electrophoresis run in the presence of 0.1 µgr/ml chloroquine. The relative positions for linear size markers are indicated to the left. The number for each lane is shown on top and the nature of each band is shown to the right. Intact DNA isolated from S. cerevisiae transformed with pYAC_MEM (lane 1); DNA isolated from E. coli cells transformed with pYAC_MEM digested with BamHI (lane 2); Intact DNA from S. cerevisiae transformed with YAC_MEM (lane 3). Hybridized with L2. D: Genomic and extra-chromosomal DNA fragments analyzed by unidirectional gel electrophoresis after digestion with BamHI and XhoI, hybridized with URA3. DNA from untransformed cells (lane 1); DNA from cells transformed with pYAC_MEM (lane 2); DNA from cells transformed with YAC_MEM (lane 3). Note the relative intensities of the genomic (chromosomal) and extrachromosomal bands in each lane.

pYAC_MEM is a 7966 bp circular minichromosome containing the following yeast elements: the bi-directional replication origin ARS4, the centromeric sequence CEN6, two tandem series of *Tetrahymena* telomeric repeats and URA3 and HIS3 genes, as indicated in inside of Figure 1A. Digestion of this circular minichromosome with BamHI (Figure 1B) releases the HIS3 containing inter-telomeric-repeats’ insert leaving the *Tetrahymena* telomeric repeats at the ends of a 6198 bp linear fragment. Yeast cells were transformed with either the circular or the linear versions of this minichromosome. DNA was isolated from selected colonies and the electrophoretic mobility of intact DNA and BamHI digested DNAs isolated from *E. coli* cells were compared (Figure 1C). To enhance the identification of topoisomers, electrophoresis was performed in the presence of 0.1 µgr/ml chloroquine. Szostak’s original observation [4] was confirmed by comparing the different electrophoretic behavior of the DNAs analyzed. While many bands with different electrophoretic mobility were detected for the circular pYAC_MEM isolated from *S. cerevisiae* in lane 1, no topoisomers were observed for the intact form of the linear YAC_MEM also isolated from *S. cerevisiae* in lane 3. This unique band, though, was slightly bigger than the single 6198 bp BamHI-pYAC_MEM digested linear fragment isolated from *E. coli* cells (lane 2). Yeast telomerase recognizes *Tetrahymena* telomeric repeats and ads yeast telomeric repeats to the chromosome ends [8] that end-up bigger (see bottom map in Figure 1B). Altogether these observations confirmed that we succeeded to obtain yeast cells transformed with circular and linear minichromosomes.

Small minichromosomes are known to be unstable in yeast [1], [3], [4]. In addition, misfunction of the centromere converts single-copy into multi-copy extrachromosomal elements [9]. To avoid cells that had copies of the minichromosomes integrated into yeast genomic DNA, we selected cells from colonies that were tested to have no or at least as few as possible minichromosome’s DNA co-migrating with high molecular weight genomic or multimeric DNAs. Experiments were performed also to determine circular and linear minichromosome’s copy number more than 30 generations after transformation. Densitometry was used to compare a single-copy chromosomal gene (URA3) with the same gene present in the extrachromosomal element. The result obtained indicated that the ratio was ∼1∶2 for circular minichromosomes and ∼1∶4 for linear YACs (see Figure 1D). In addition, mitotic stability was measured in cells growing over 17 generations in non-selective medium [10]. The results obtained indicated that linear YACs were retained in approximately 50% of the colonies. Altogether, these experiments indicated that although centromeres appeared less efficient in linear as compared to circular minichromosomes, a significant proportion of cells retained extra-chromosomal single copies of both pYAC_MEM and YAC_MEM.

**Figure 2:**
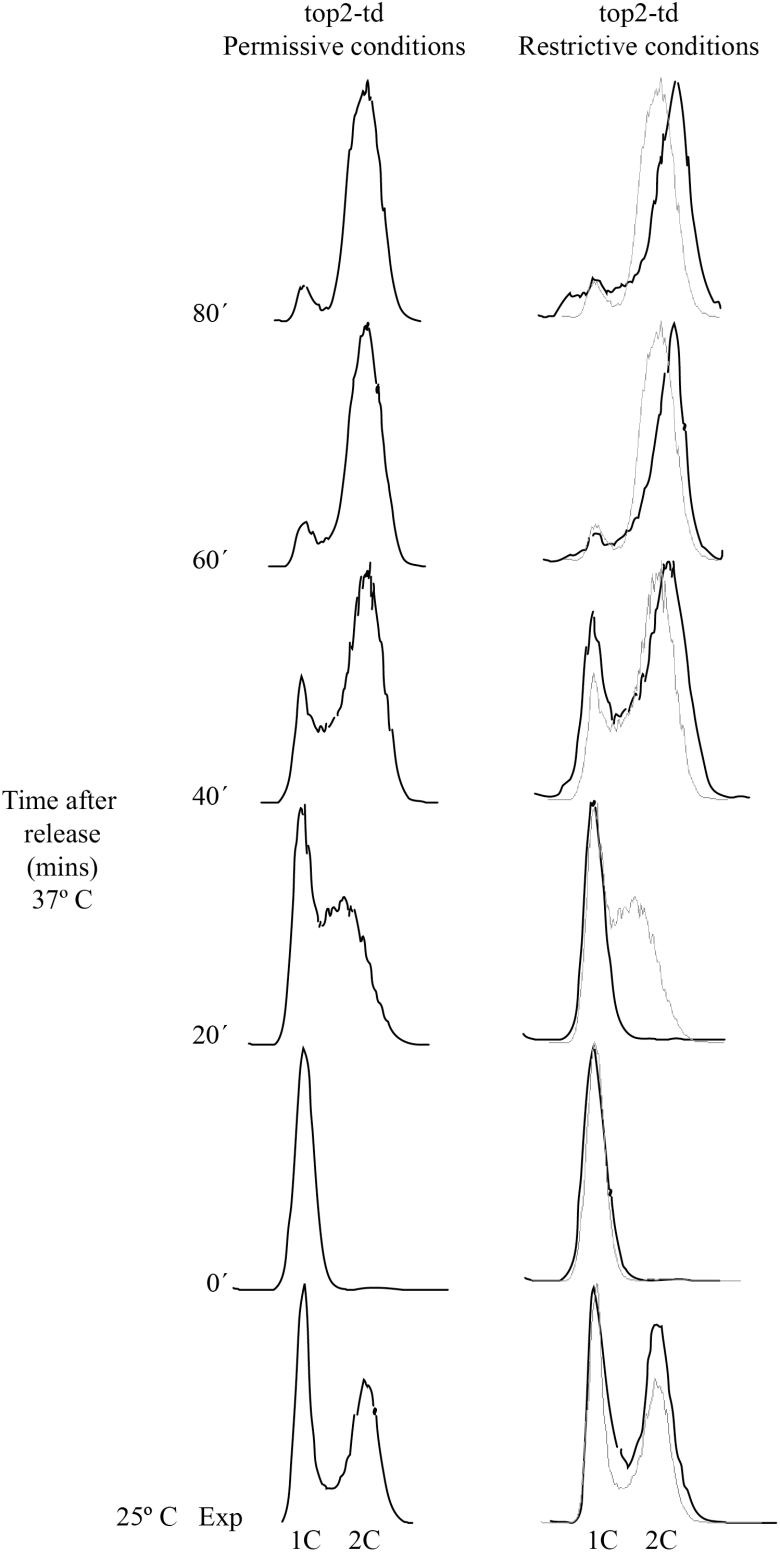
Cell synchronization and monitor of their synchronous progression through the S-phase with and without DNA topoisomerase 2. top2-td cells transformed with the circular minichromosome pYAC_MEM were synchronized at the G1-S boundary and released synchronously into the S-phase at either permissive or restricted conditions. Samples were taken at regular intervals, the cells stained with SYTOXGreen and analyzed by flow cytometry. The data corresponding to fluorescence-activated cell sorting analysis of DNA content is shown. For comparison, top2-td curves from cells grown at the permissive temperature are indicated in pale gray for the diagrams of cells grown at the restrictive conditions.

We wanted to study the replication intermediates (RIs) and segregation products of pYAC_MEM and YAC_MEM with and without DNA topoisomerases. To this end we used both minichromosomes to transform a top2-td degron strain [11] (provided by Jonathan Baxter). Cells were synchronized at the G1-S boundary with α-factor and released into the S-phase at the permissive or restricted conditions (see material and methods). Cell aliquots were fixed from exponentially growing cultures and at different times after their release into the S-phase. They were stained with SYTOXGreen and analyzed by flow cytometry (Figure 2). The results obtained confirmed previous observations [12] indicating that in the presence of DNA topoisomerases (top2-td cells grown at permissive conditions) cell progression into S-phase was already detected at the 20 min sample and 2C cells progressively accumulated thereafter. In the absence of topoisomerase 2 (top2-td cells grown at the restrictive conditions) a delay occurred in the entry of cells into S-phase. However, these cells recovered fast and no significant difference between these cells and those growing at the permissive conditions was observed by 40 min after the release. Note that by 80 min, cells growing at the restrictive conditions showed DNA contents slightly bigger than those cells growing at the permissive conditions. This is due to errors in cytokinesis that leads to multinucleated cells [12]. These experiments confirmed that the transformed yeast cells were able to complete S-phase in the absence of topoisomerase 2. They were not able to divide properly, though, because sister chromatids remained catenated [7], [12].

### Analysis of replication products of pYAC_MEM and YAC_MEM with and without DNA topoisomerases

**Figure 3:**
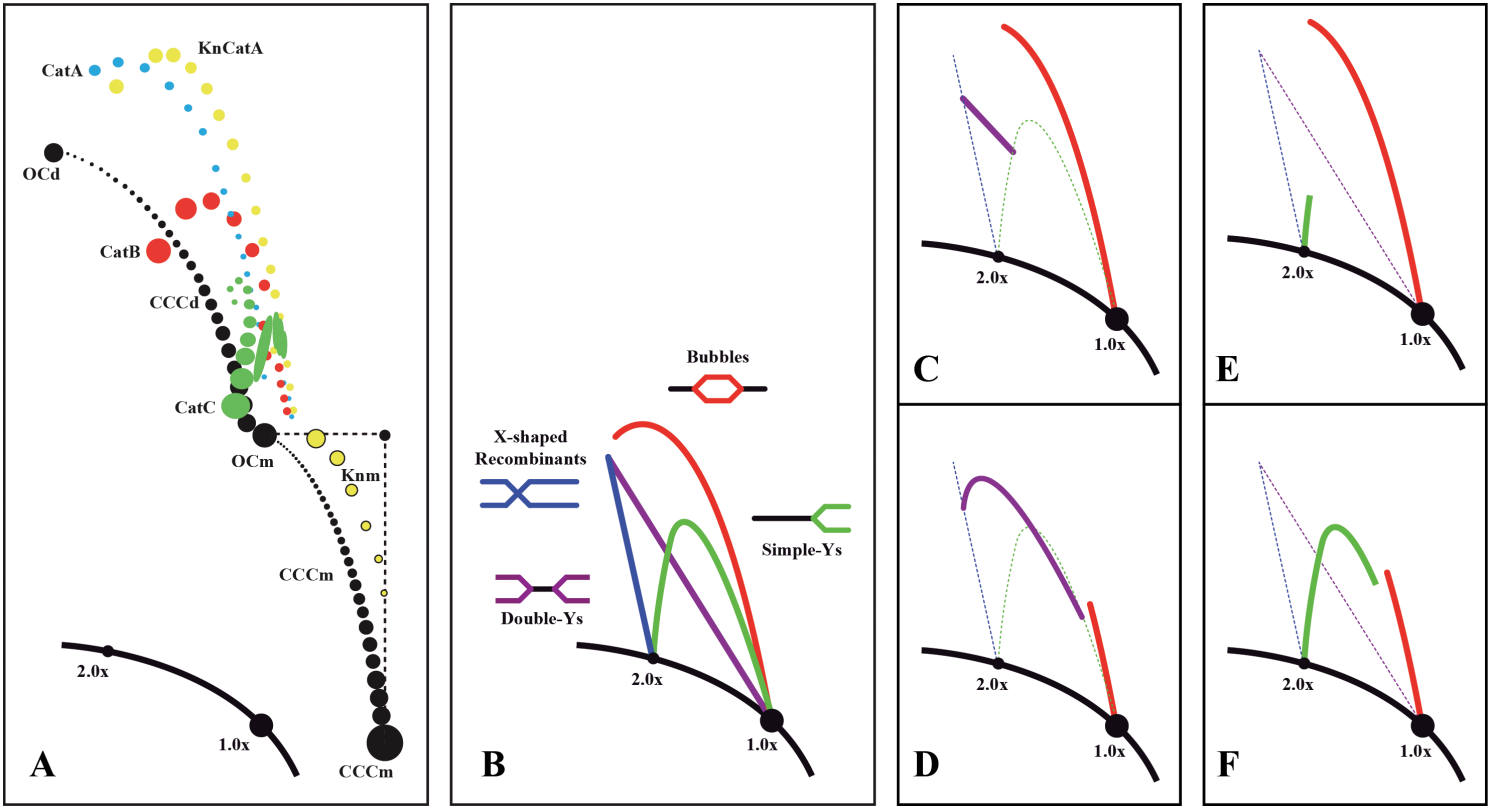
Cartoons illustrating the different patterns generated by the stereoisomers of undigested circular minichromosomes and linear DNA replication intermediates in 2D gels. A: Linear forms (1.0x and 2.0x) and covalently closed monomers (CCCm) and dimers (CCCd) are depicted in black. CatA (catenanes where both rings are nicked) are depicted in light blue. CatB (catenanes where one ring is nicked and the other covalently closed) are depicted in red. CatC (catenanes where both rings are covalently closed) are depicted in green. KnCatA (nicked-catenanes where one or both rings are knotted) are depicted in yellow. And knotted monomers (Knm) are depicted in black encircled yellow. B: Complete patterns generated by linear replication intermediates. Bubbles in red, Simple-Ys in green, Double-Ys in violet, X-shaped recombinants in blue and unreplicated linear fragments in black. C: Patterns illustrating the transition from Bubbles to Double-Ys when it occurs after 1.5x. D: Patterns illustrating the transition from Bubbles to Double-Ys when it occurs before 1.5x. Note that here Double-Ys have a characteristic inflection. E and F: Patterns illustrating transitions from Bubbles to Simple-Ys.

**Figure 4:**
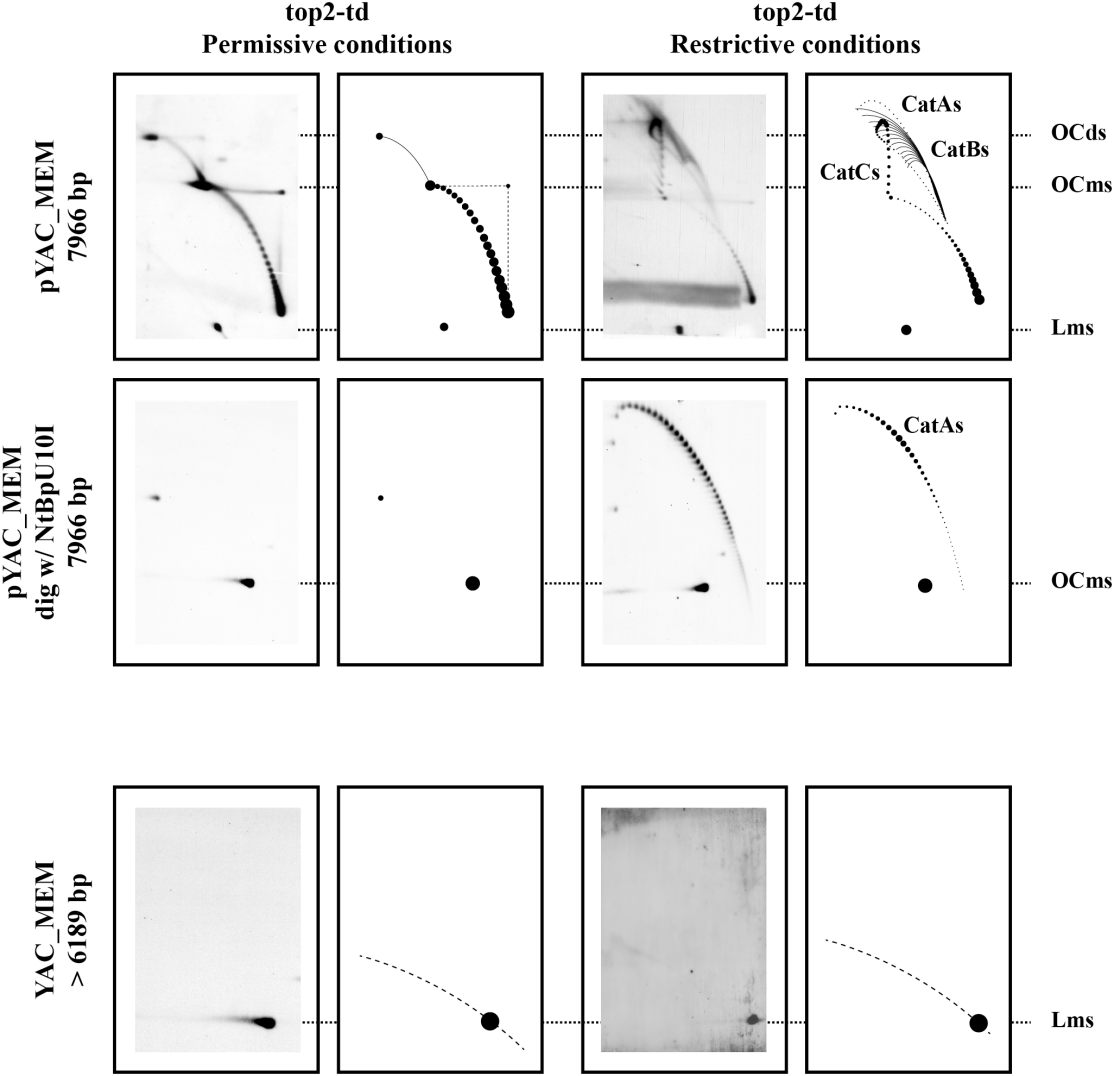
Analysis of replication products of pYAC_MEM and YAC_MEM with and without DNA topoisomerases. Synchronized top2-td cells transformed with either pYAC_MEM or YAC_MEM were fixed 80 minutes after their release into the S-phase under permissive or restricted conditions. Undigested pYAC_MEM, the same DNA digested with the nicking enzyme NtBpU10I and undigested YAC_MEM DNAs were analyzed in 2D gels. The corresponding immunograms are shown together with a diagrammatic interpretation of the most prominent signals to their right. Cats  =  Catenanes A, B and C; OCms  =  Monomer Open Circles; Lms  =  Monomer Linears.

It was previously shown that top2-td cells synchronized with α-factor complete S phase ∼60 minutes after their release [12]. To examine minichromosome’s segregation, cells were fixed 80 min after their release (see Figure 2). DNA was isolated and analyzed undigested in 2D gels. The cartoons shown in Figure 3 illustrate the different patterns expected when undigested circular molecules and linear DNA replication intermediates are analyzed in two-dimensional (2D) agarose gel electrophoresis [13]–[18]. The results obtained are shown in Figure 4. For pYAC_MEM under permissive conditions, monomeric and dimeric topoisomers were clearly distinguished (left top panel) suggesting that topoisomerase 2 efficiently decatenated sister duplexes once replication was over. On the contrary, under restricted conditions, in addition to monomeric topoisomers, catenanes (CatAs, CatBs and CatCs) accumulated (right top panel), indicating that here topoisomerase 2 was inactive [12]. To confirm the latter observation, DNA was digested with the nicking endonuclease NtBpU10I prior to analysis in 2D gels. Only linear monomers and dimers were detected for the DNA of cells released under permissive conditions (left mid panel), whereas CatAs were the most prominent signal detected for the DNA of cells released under restricted conditions (right mid panel). Surprisingly, for the linear YAC_MEM, the only signal detected corresponded to linear monomers (third row panels) regardless of whether the cells were released into S-phase at the permissive or restricted conditions. These observations strongly suggested that linear YACs were able to segregate in the absence of Topo 2. Note that herein segregation is used to indicate that after replication, sister duplexes are are not catenated and are able to separate from each other. In other words, here segregation is not used to indicate errors in spindle formation and unimpaired mitosis.

### Analysis of the replication intermediates of pYAC_MEM

**Figure 5:**
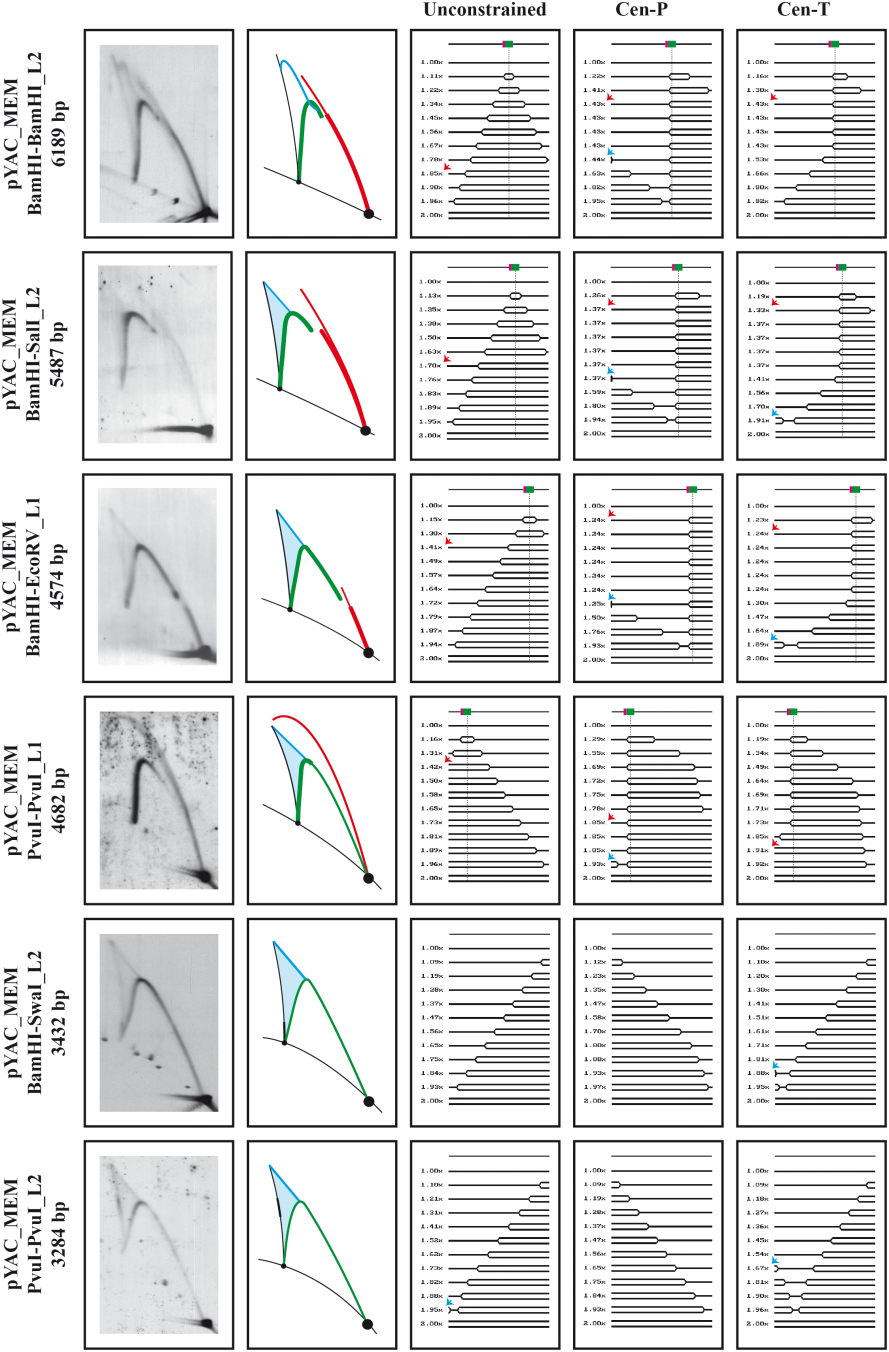
Analysis of the replication intermediates of pYAC_MEM. Synchronized top2-td cells transformed with pYAC_MEM were fixed 40 minutes after their release into the S-phase. The DNA was isolated, digested with the restriction enzymes shown to the left and analyzed in 2D gels. The corresponding immunograms are shown in the far left column with their corresponding interpretative diagrams to their right. Bubble arcs in red, simple-Y arcs in green and double-Ys in pale blue. Linear molecules and recombinants are shown in black. The simulation program 2D gel [19] was used to predict the shape of twelve consecutive RIs if replication proceeds unconstrained or if the leftward moving fork stalls permanently (Cen-P) or transiently (Cen-T) at the centromere CEN6. A linear map is shown on top of each series of RIs showing the relative positions of ARS4 (in green) and CEN6 (in magenta). The relative masses of the RIs are shown to the left. Red arrows indicate the transition mass of the RIs from bubbles to simple-Ys whereas blue arrows indicate transition mass from simple-Ys to double-Ys.

We wanted to confirm that minichromosomes were able to replicate at the molecular level. To characterize their replication mode, top2-td transformed cells were fixed at different times after their release into the S-phase and the corresponding RIs were examined in 2D gels after linearization with a number of different restriction enzymes (see Figures 1A and B). RIs were unambiguously detected in the samples collected between 20 and 60 minutes after their release. The 2D gel patterns obtained were similar although the strength of the signals varied. The strongest signals for RIs were obtained 40 minutes after the release. For pYAC_MEM the results obtained are shown in Figure 5. Digestion of the circular minichromosome with BamHI and hybridization with L2 (Figure 1A and top panel in B) allowed us to examine the 2D gel patterns generated by RIs of the larger 6198 bp resulting fragment containing ARS4 and CEN6 (top panel in Figure 5). The simulation program 2D gels [19] was used to predict the shape of the RIs responsible for the patterns observed. None of the patterns detected corresponded to those expected for unconstrained replication of the circular minichromosome. In such a case initiation would occur at ARS4 in a bi-directional manner. The shapes of the RIs predicted for this model were called “Unconstrained” in Figure 5. Note that transition from bubbles to simple-Ys would have occurred at a mass of ∼1.8x. The simulation program predicted that the patterns observed corresponded to two different replication models that co-existed. In the first one, here called Cen-P, initiation of DNA replication occurs at ARS4 in a bi-directional manner. The replication fork moving counterclockwise, though, stalls permanently at CEN6. The clockwise moving fork, on the other hand, progresses all around the circular minichromosome to encounter the stalled fork at CEN6. Progression of this clockwise moving fork might not be uniform, though, as it could slow-down variably [20], [21]. Note that transition from bubbles to double-Ys for this replication model would occur at a mass of ∼1.4x. In addition, as more than 50% of the RIs would be double-Ys, the corresponding double-Y arc would show an inflection [16], [22]. The second replication model (here called Cen-T) predicted by the simulation program 2D gels [19] assumed that initiation of DNA replication also occurs at ARS4 in a bi-directional manner. In this model, though, the fork moving counterclockwise stalls at CEN6 only transiently and continues its way after variable periods of time. This transient blockage allows progression of the other clockwise moving fork to variable lengths and termination (the head-on encounter of both forks) would occur at different sites in different cells. Since electrophoretic mobility of RIs in agarose gels is not linear, the signal generated by infrequent different double-Ys would only be detected faintly for molecules showing high electrophoretic mobility. Otherwise, the double-Ys would generate a triangular smear [16], [22]. Note that here transition from bubbles to simple-Ys would also occur at a mass of ∼1.4x.

To confirm the replication models called Cen-P and Cen-T predicted by the 2D gel simulation program [19], DNA isolated from synchronized top2-td cells transformed with pYAC_MEM were harvested 40 minutes after their release into S-phase, digested with different restriction enzymes that placed CEN6 at different relative positions in the linearized fragment, and analyzed in 2D gels. The different circular and linear maps are shown in Figures 1A and B and the corresponding immunographs are shown in Figure 5. Note that for the BamHI-SalI 5487 bp fragment hybridized with L2 (second panel in Figure 5), both replication models (Cen-P and Cen-T) predicted the transition from bubbles to simple-Ys would occur at a mass or ∼1.3x. For the BamHI-EcoRV 4574 bp fragment hybridized with L1 (third panel in Figure 5), both replication models predicted the transition from bubbles to simple-Ys would occur at a mass or ∼1.2x. For the PvuI-PvuI 4682 bp fragment hybridized with L1 (fourth panel in Figure 5), as here ARS4 would be placed close to the left end of the fragment, both models predicted RIs containing an internal bubble would occur up to a mass of ∼1.9x. The immunographs and their interpretations (second, third and fourth panels in Figure 5) confirmed the replication models predicted by the simulation program 2D gels [19]. Finally, for the BamHI-SwaI 3432 bp and the PvuI-PvuI 3284 bp fragments lacking ARS4, the models predicted the RIs would mostly consist of simple-Ys and only the Cen-T model predicted some termination events would take place at different relative positions close to the left end of both fragments. Indeed, the corresponding immunographs showed stronger signals on the arc of X-shape recombinants, suggesting termination events at the predicted locations (see the two bottom panels in Figure 5). Altogether these observations confirmed that in pYAC_MEM replication forks stall at the centromere CEN6 either in a permanent or a transient manner [23]. It should be noted that each immunograph in Figure 5 revealed a mixture of different populations that replicated mainly according to the two models predicted by the program.

**Figure 6:**
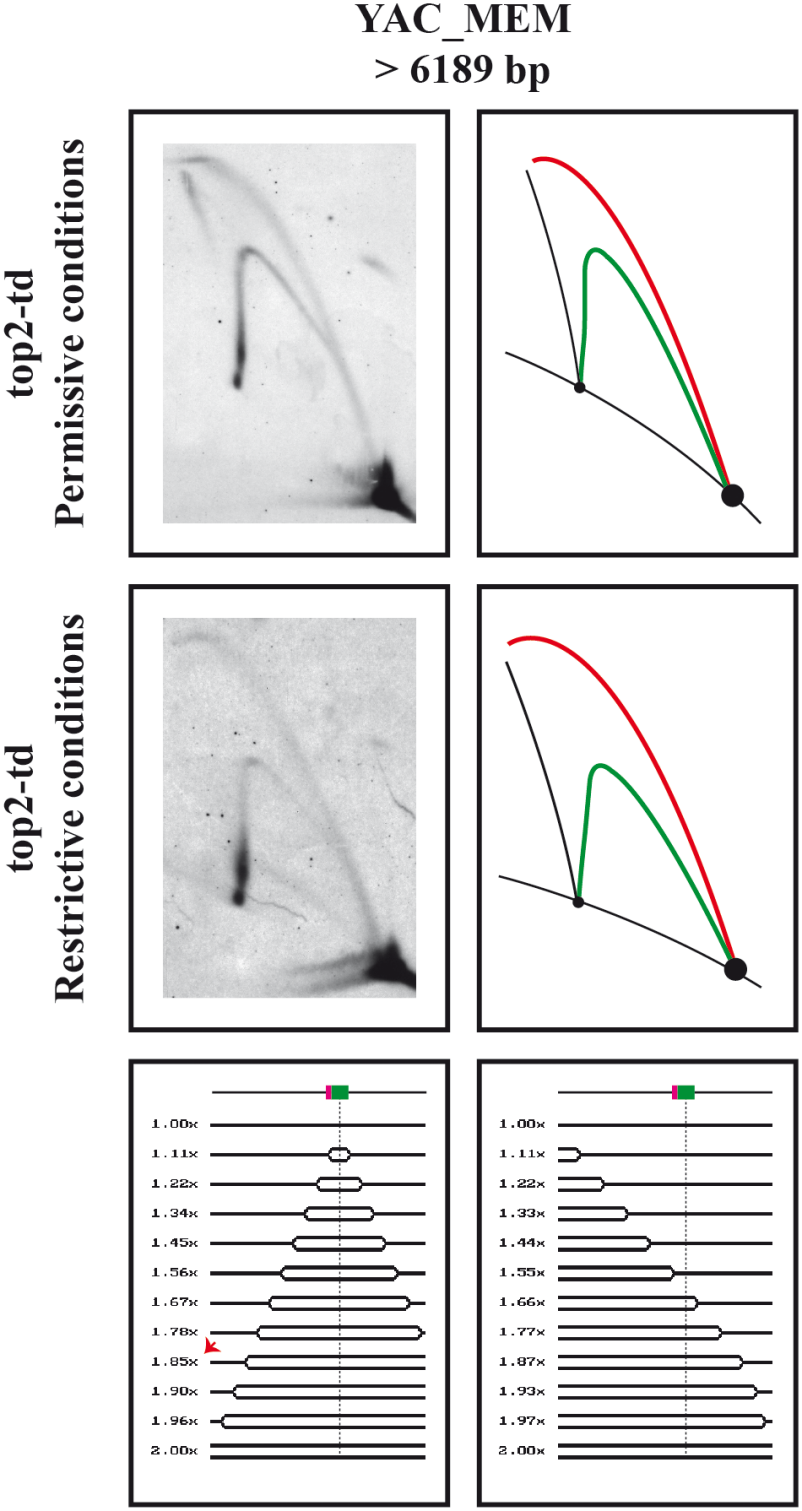
Analysis of the replication intermediates of YAC_MEM in the presence and absence of DNA topoisomerases. Synchronized top2-td cells transformed with YAC_MEM were fixed 40 minutes after their release into the S-phase under permissive or restricted conditions. The DNA was isolated and analyzed undigested in 2D gels. The corresponding immunograms are shown with their corresponding interpretative diagrams to their right. Bubble arcs in red and simple-Y arcs in green. Linear molecules and recombinants are shown in black. The simulation program 2D gel [19] was used to predict the shape of twelve consecutive RIs if replication initiated bi-directionally at ARS4 and proceeds unconstrained (shown to the left) or if initiation occurs at one telomere (shown to the right). A linear map is shown on top of each series of RIs showing the relative positions of ARS4 (in green) and CEN6 (in red). The relative masses of the RIs are shown to the left. The red arrow indicates the transition from bubbles to simple-Ys.

To find out whether or not the replication of linear minichromosomes follows a similar pattern, cells transformed with YAC_MEM were synchronized at the G1-S boundary, released into S-phase at the restrictive conditions and harvested 40 minutes thereafter (see Figure 2). In this case, though, only undigested RIs were analyzed in 2D gels. The results obtained are shown in Figure 6. In both cases: with topoisomerase 2 (top2-td cells grown at the permissive temperature) and without topoisomerase 2 (top2-td cells grown at the restrictive temperature), the replication patterns observed in the immunograms were identical. Surprisingly, an almost complete bubble arc and another complete simple-Y arc were clearly detected. The signal for the simple-Y arc was stronger toward the end of replication as the mass of RIs approached 2.0x. This does not necessarily indicate fork stalling as it could be simply due to the juxtaposition of both replication patterns in this region as predicted by the simulation program 2D gels [19]. Note that some linear YACs initiated replication at ARS4 in a bi-directional manner and both replication forks progressed unconstrained. In these molecules transition from bubbles to simple-Ys occurred at a mass ∼1.8x the mass of unreplicated molecules. On the other hand, the detection of a complete simple-Y arc indicated that in some linear YACs initiation of replication also occurred at the telomeres.

### Construction of pYAC_MEM_RFB+ and YAC_MEM_RFB+ and analysis of their replication intermediates

**Figure 7:**
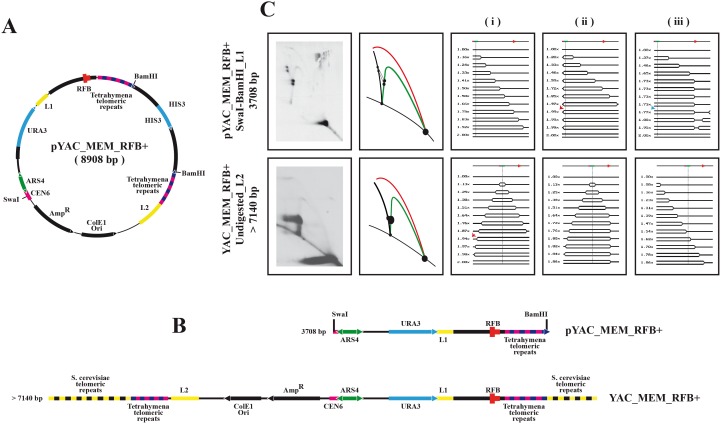
Construction of pYAC_MEM_RFB+ and YAC_MEM_RFB+ and analysis of their replication intermediates. A: Name, mass and genetic map of the circular minichromosome. The relative positions of its most relevant features are indicated inside: The centromeric sequence CEN6, the autonomous replication sequence (ARS4), URA3, the lambda DNA marker sequence (L1), the ribosomal RFB, *Tetrahymena* telomeric repeats, HIS3, the lambda DNA marker sequence (L2), the ColE1 unidirectional origin (ColE1 Ori) and the ampicillin-resistance gene (AmpR). Outside, the relative positions of sites recognized by specific restriction endonucleases are indicated. B: The corresponding linear map of the restriction fragment used and its size. Below, the genetic map of YAC_MEM_RFB+. C: Synchronized top2-td cells transformed with either pYAC_MEM_RFB+ or YAC_MEM_RFB+ were fixed 40 minutes after their release into the S-phase. The DNA was isolated, digested with the restriction enzymes shown to the left or kept undigested and analyzed in 2D gels. The corresponding immunograms are shown at the far left column with their corresponding interpretative diagrams to their right. Bubble arcs in red and simple-Y arcs in green. Linear molecules, recombinants and accumulated forms are shown in black. The simulation program 2D gel [19] was used to predict the shape of twelve consecutive RIs. For the BamHI-SwaI restriction fragment of the circular minichromosome pYAC_MEM_RFB+, if replication initiates at ARS4 and both forks proceed unconstrained (i), if replication initiates at ARS4, the leftward moving fork stalls permanently at CEN6 and the rightward moving fork moves unconstrained through the RFB (ii), and if replication initiates at ARS4, the leftward moving fork stalls transiently at CEN6 and the rightward moving fork stalls permanently either at the first or the second closely spaced sites of the RFB (iii). Below, for the linear minichromosome YAC_MEM_RFB+ on top, if replication initiates at ARS4 and both forks proceed unconstrained (i), if replication initiates at ARS4 and the rightward moving fork stalls permanently at the RFB (ii) and if replication initiates at the left telomere and the rightward moving fork stalls permanently at the RFB (iii). A linear map is shown on top of each series showing the relative positions of ARS4 (in green), CEN6 (in magenta) and the RFB (in red). The relative masses of the RIs are shown to the left. Red arrows indicate the transition mass of the RIs from bubbles to simple-Ys whereas blue arrows indicate the transition mass from simple-Ys to double-Ys.

Several DNA sequences that form secondary structures or bind protein complexes are known barriers to replication and potential inducers of genomic instability [24], [25]. To find out if in addition to centromeres, other natural barriers (RFBs) also depend on DNA supercoiling, we cloned a DNA fragment containing the yeast ribosomal RFB [26]–[28] into pYAC_MEM in its active orientation. The yeast ribosomal DNA sequence called RFB located in the intergenic spacer binds the protein called FOB1 and this complex stalls replication forks in a polar manner [29], [30]. The map of the resulting minichromosome (pYAC_MEM_RFB+) is shown in Figure 7A. Digestion of this circular minichromosome with BamHI (Figure 7B) releases the HIS3 containing inter-telomeric-repeats’ insert leaving the *Tetrahymena* telomeres at the ends of a 7140 bp linear fragment. Top2-td cells were transformed with either the circular or the linear form of this minichromosome, blocked at the G1-S boundary with α-factor and released synchronously into the S-phase. Cells were harvested 40 minutes after the release and the RIs analyzed in 2D gels. For the circular pYAC_MEM_RFB+, DNA was digested with BamHI and SwaI and hybridized with L1 as indicated in the top panels of Figure 7A and B. For the linear YAC_MEM_RFB+, DNA was analyzed undigested and hybridized with L2 (see bottom panel in Figure 7B). The results obtained demonstrated that the yeast ribosomal RFB stalled replication forks in both minichromosome forms: circular and linear (Figure 7C).

## Discussion

### Topo 2 is dispensable for transcription, replication and segregation of small linear YACs

Supercoiling is thought to play a crucial role in transcription and replication. Negative supercoiling is thought to assist the binding of factors required to start transcription and replication [31] and topoisomerases 1 and 2 act within a 600 bp region spanning the replicating forks although their independent ablation does not affect fork progression [32]. At elongation, as positive supercoiling accumulates ahead of progressing forks, topoisomerases are needed to maintain DNA under negative superhelical strain and thus facilitate unwinding. In addition, during replication swiveling of the progressing fork might allow some positive supercoiling to diffuse back behind the fork where it takes the form of pre-catenanes [33]. Here again Topo 2 is required to eliminate them and the resulting catenanes to allow segregation of the sister chromatids once replication is over. Topoisomerase 3 together with the RecQ helicase are able to decatenate DNA in *in* *vitro* assays [34]–[36] but for circular minichromosomes catenated duplexes accumulate in the absence of Topo 2 *in* *vivo* [7], [12] and results in Figure 4). It was recently shown that DNA supercoiling (negative in prokaryotes and positive in eukaryotes) facilitates decatenation [12], [37]. Small linear YACs are not supercoiled [1,3,4 and results here described], but they transcribe, replicate and segregate regardless of Topo 2. Cells transformed with YAC_MEM would not be able to survive unless the URA3 gene of the small linear minichromosome remains fully operative. Cohesin was found to hold together sister chromatids even after complete decatenation by Topo 2 [38] but this complex is not able to decatenate sister duplexes by itself. Altogether, our observations suggest that DNA supercoiling could modulate but is not essential for transcription, replication and segregation.

### DNA supercoiling might play a key role in the modulation of replication fork progression

The antagonism between centromeres and functional telomeres might cause centromere’s misfunction [9]. This would explain why replication forks progressed unconstrained throughout centromeric DNA in linear YACs but not in the circular version [39]. Alternatively, DNA supercoiling might play a significant role for centromeres to hinder replication fork progression. Other barriers, such as the yeast ribosomal RFB, which is known to work in extra-chromosomal circular minichromosomes [25]–[30], worked fine regardless of DNA topology (See Figure 7).

### DNA torsional tension dissipates freely throughout the telomeres

It was recently shown that in *S. cerevisiae* an excess of positive supercoiling produces a general impairment of transcription initiation except for genes situated at <100 kb from the chromosomal ends [5]. This observation led the authors to suggest that DNA helical tension dissipates at chromosomal ends. The fine structure of telomeres is still imperfectly understood [40]. The presence of a 3′ single-stranded G-rich overhang appears unquestionable [41], but the formation of t-loops [42], [43] and/or G-quartets [44] are still under debate [45]. In any case, the results obtained here indicate that the putative t-loops and/or G-quartets at the end of eukaryotic chromosomes are not topological barriers during replication or they could form transiently [46]. The latter observation might fit with the idea that the related helicases pif1 and/or rrm3 [47], [48] could play a crucial role in the dissipation of supercoiling through the telomeres *in* *vivo*. In any case, our results support the idea that supercoiling dissipates throughout the telomeres. This would explain why small linear YACs appear relaxed and need no topoisomerases to transcribe, replicate and segregate.

### Initiation of DNA replication can occur at the telomeres

The detection in 2D gels of a simple-Y arc among the replication intermediates of small linear YACs indicated that a single fork that progresses unconstrained from one end to the other could complete their replication. This observation implies that in some cases initiation of DNA replication occurs at the telomeres. Similar observations have been reported for telomeric DNA in *Xenopus* cell free extracts [49] and specific telomeres of individual human chromosomes in embryonic stem (ES) cell lines and two primary somatic cell types [50]. No bubble arc was observed for the 3284 bp PvuI-PvuI DNA fragment hybridized with L2 (see Figures 1 and 5), indicating that replication does not initiate at telomeric repeats in the circular pYAC_MEM.

### Telomeres and DNA topoisomerases might have co-evolved

The origin and evolution of DNA topoisomerases have been reviewed elsewhere [51]–[53]. The observation that the main families of topoisomerases are not homologous, suggests they originated independently in the three cellular domains: prokaryotes, archaea and eukaryotes. Although the need for topoisomerases was already recognized by Watson and Crick as soon as they proposed their model for the structure of DNA [54], it seems likely that they were not needed for the first forms of life on earth, as proposed by the so-called LUCA (Last Universal Common Ancestor) model with an RNA genome without the need for DNA topoisomerases [51]. Interestingly, it was recently shown that in the filamentous bacteria of the genus *Streptomyces*, Topoisomerase IV, the prokaryotic decatenase, is required for partitioning of their circular chromosomes but not the linear ones [55]. In any case, to our knowledge the data presented here represents the first experimental evidence where it is clearly shown that even in modern eukaryotes, DNA replication and segregation of small linear YACs can occur in the absence of DNA topoisomerases.

The observations we reported here advance many new questions: At which size of a YAC do topoisomerases become essential? Which is the minimal distance for two ARSs to avoid interference in a YAC? In such a YAC, would Topo 2 be needed to resolve the entanglements generates as two forks moving in opposite directions approach each other? Could a YAC with an internal topological domain and two open telomeric domains be considered a “real” eukaryotic chromosome prototype? These are the type of questions we are currently trying to answer.

## Methods

### Yeast strains

Yeast strains were based on W303-1a (MATα ade2-1 ura3-1 his3-11, trp1-1 leu2-3, can1-100) modified for use with the “degron” system (strain YST114), as described [56] supplied by Jonathan Baxter.

### Media and cell cycle synchronization

Top2-td cells were grown at 25°C in synthetic medium without uracil containing 2% raffinose (Raf). Cultures were transferred to complete medium YP with Raf until midlog and yeast cells were arrested at G1 with 10 µg/ml α-factor. Induction of the Ubr1 promoter was achieved by addition of 2% galactose. When 90% of cells were in G1 (120–150 min) 50 µg/ml Doxycycline was added. After 30 min the cultures were shifted to 37°C for 1∶30 hour. Cells were washed 4 times and incubated in YP medium plus 2% Raf, 2% gal, and 50 µg/ml doxyclycline at 37°C and cell samples were taken at the indicated times. Time 0 was defined as the time of the first wash. Cell synchronization was performed as described elsewhere [12].

### Flow cytometry

Samples for flow-cytometric analysis were collected and processed as described before [47] and analyzed using a XL Coulter from Beckman Coulter.

### DNA preparation

DNA was isolated by the Hoffman method [57]. In the case of replication intermediates, DNA was prepared according to Huberman’s procedure [58] with some modifications.

### DNA treatments

DNA was digested with BamHI, EcoRV, KpnI, NsiI, PvuI, PvuII, SalI, SwaI, XhoI, (New England Biolabs) and Nt.Bpu10I (Thermo Scientific) for at least 2 hours at 37°C except for SwaI that was incubated at 25°C.

### 2D agarose gel electrophoresis and southern transfer

The first dimension was in a 0.35–0.5% agarose gel (Seakem; FMC Bioproducts) in TBE buffer (89 mM Tris-borate, 2 mM EDTA) at 0.9 V/cm at room temperature for 27–38 h. The second dimension was in a 0.9–1.2% agarose gel in TBE buffer and was run perpendicular to the first dimension. The dissolved agarose was poured around the excised agarose lane from the first dimension and electrophoresis was at 4.5 V/cm in a 4°C cold chamber for 11–13 h. in the presence of 0.3 µg/ml ethidium bromide when necessary. Southern transfer was performed as described before [20], [59].

### Non-radioactive hybridization

DNA probes were labelled with digoxigenin using the DIG-High Prime kit (Roche). Membranes were pre-hybridized in a 20 ml pre-hybridization solution (2x SSPE, 0.5% Blotto, 1% SDS, 10% dextran sulphate and 0.5 mg/ml^−1^ sonicated and denatured salmon sperm DNA) at 65°C for 4–6 h. Labeled DNA was added and hybridization lasted for 12–16 h. Hybridized membranes were sequentially washed with 2x SSC and 0.1% SDS at room temperature for 5 min twice and with 0.1x SSC and 0.1% SDS at 68°C for 15 min twice as well. Detection was performed with an antidigoxigenin-AP conjugate antibody (Roche) and CDP-Star (Perkin Elmer) according to the instructions provided by the manufacturer. Quantification of autoradiograms was performed using a ImageJ64 software.

### Construction of yeast replicating plasmid

pYAC_MEM (7966 bp): First, two lambda sequences of 273 pb and 436 pb, named L1 and L2, were inserted, respectively, between SalI and KpnI of pRS316 to use them as DNA marker sequences and pRS316 was converted into a 4788 bp centromeric plasmid called pRSF1_MEM. Second, the F1 origin and LacZ gene were removed with Nsil and the ends were ligated to yield pRS_MEM. Third, the XhoI fragment of the circular chromosome pYAC_RC [60], that contains the 3163 bp telomeric sequences of Tetrahymena thermophile [61] separated by sequence with histidine gene, were inserted into the XhoI site of pRS_MEM to obtain pYAC_MEM.

pYAC_MEM_RFB+ (8908 bp): The pBB6-RFB+ [62] was digested with EcoRI to isolate the RFB fragment which was inserted into the SalI site of the pYAC_MEM.

Both circular minichromosomes were linearized with BamHI to obtain the linear forms.

Minichromosomes were introduced into yeast by lithium acetate method [63].
